# Activated γδ T Cells With Higher CD107a Expression and Inflammatory Potential During Early Pregnancy in Patients With Recurrent Spontaneous Abortion

**DOI:** 10.3389/fimmu.2021.724662

**Published:** 2021-08-17

**Authors:** Long Yu, Yang Zhang, Jinfeng Xiong, Jianjun Liu, Ying Zha, Qi Kang, Pan Zhi, Qiang Wang, Hui Wang, Wanjiang Zeng, Yafei Huang

**Affiliations:** ^1^Department of Immunology, Medical College, Wuhan University of Science and Technology, Wuhan, China; ^2^Department of Pathogen Biology, School of Basic Medicine, Tongji Medical College, Huazhong University of Science and Technology, Wuhan, China; ^3^Department of Obstetrics and Gynecology, Tongji Hospital, Tongji Medical College, Huazhong University of Science and Technology, Wuhan, China; ^4^Department of Obstetrics and Gynecology, The Second Hospital of Chaoyang City, Chaoyang, China

**Keywords:** γδ T cells, recurrent spontaneous abortion (RSA), PD1, CD107a, IL-17A

## Abstract

Previous studies have reported the involvement of γδ T cells in recurrent spontaneous abortion (RSA); however, both pathogenic and protective effects were suggested. To interrogate the role of γδ T cells in RSA, peripheral blood from RSA patients and healthy women with or without pregnancy were analyzed for γδ T cells by flow cytometry (*n* = 9–11 for each group). Moreover, the decidua from pregnant RSA patients and healthy controls (RSA-P and HC-P group, respectively) was simultaneously stained for γδ T cells by immunohistochemistry (IHC) and bulk sequenced for gene expression. Our results demonstrated that the frequencies of peripheral γδ T cells and their subpopulations in RSA patients were comparable to that in healthy subjects, but the PD1 expression on Vδ2^+^ cells was increased in pregnant patients. Furthermore, peripheral Vδ2^+^ cells in RSA-P patients demonstrated significantly increased expression of CD107a, as compared to that in pregnant healthy controls. In addition, RSA-P patients had higher proportion of IL-17A-secreting but not IL-4-secreting Vδ2^+^ cells compared to the control groups. In decidua, an inflammatory microenvironment was also evident in RSA-P patients, in which *CCL8* expression and the infiltration of certain immune cells were higher than that in the HC-P group, as revealed by transcriptional analysis. Finally, although the presence of γδ T cells in decidua could be detected during pregnancy in both RSA patients and healthy subjects by multicolor IHC analysis, the expression of CD107a on γδ T cells was markedly higher in the RSA-P group. Collectively, our results indicated that the increased activation, cytotoxicity, and inflammatory potential of peripheral and/or local γδ T cells might be responsible for the pathogenesis of RSA. These findings could provide a better understanding of the role of γδ T cells in RSA and shed light on novel treatment strategies by targeting γδ T cells for RSA patients.

## Introduction

With an incidence of 1%–3% worldwide (1, 2), RSA was defined as two or more consecutive pregnancy losses prior to the 20th week of gestation. Although many causes have been implicated in the occurrence of RSA, such as abnormal chromosome, endocrine dyscrasia, reproductive malformation, and infection, the etiology of RSA is still elusive; approximately 50% of RSA is diagnosed without specifically defined cause of abortion (3) and is therefore called unexplained RSA (URSA). Nonetheless, increasing evidence has suggested that URSA likely results from local (i.e., maternal–fetal interface) or systemic immune disorder.

Pregnancy is an immunological enigma wherein semi-allogenic fetal antigens are present at the feto-maternal interface. Therefore, maternal immune system was originally hypothesized to be “inert” or “suppressive” during gestation to secure the success of pregnancy, as proposed by Sir Peter Medawar in 1953 (4). However, current data suggest that the successful pregnancy requires an active, robust, dynamic, and tightly regulated immune system across the three stages of pregnancy (5). In the first trimester, maternal immune system is better pro-inflammatory to allow the successful implantation and placentation, and it must be subsequently switched to be anti-inflammatory to support fetal growth during the second trimester. Finally, in the third trimester, a second inflammatory response is necessary for the initiation of parturition. In this scenario, multiple immune cells have to coordinate to create an optimal immune milieu both at the maternal–fetal interface and in periphery for the success of pregnancy. On the contrary, the dysregulation of peripheral or local immune system might lead to the occurrence of RSA. To date, changes of NK cells, macrophages, neutrophils, and αβ T cells during heathy pregnancy and RSA have been extensively investigated (6); however, the role of γδ T cells remains less characterized.

As one of the three lymphocyte populations that express clonally distributed antigen receptors, γδ T cells, unlike αβ T cells and B cells of adaptive immune system, often possess a pre-activated phenotype in the steady state (7). In contrast to αβ T cells that recognize peptide antigens presented by MHC molecules (pMHC complex), γδ T cells are instead capable of reacting to a variety of antigens ranging from free peptides, lipids, phosphoantigens, and intact proteins such as MHC-I-like molecules, to pMHC complex (8, 9). Upon antigen recognition and stimulation, γδ T cells can be further activated and exert multiple functionalities, including cytotoxicity, interaction with other immune cells, and antigen presentation to αβ T cells (10, 11). Therefore, γδ T cells are considered to be the bridge between innate and adaptive immunity and have an important role in health and diseases. The role of γδ T cells in pregnancy has been suggested since 1992, when Born and colleagues found that reproductive tract γδ T cells are increased nearly 100-fold in pregnant animals compared with nonpregnant animals (12). Simultaneously, Mincheva-Nilsson et al. identified a high number of γδ T cells in the human decidua during early pregnancy (13), which was confirmed by subsequent studies (14, 15), implying the important role of γδ T cells in pregnancy. To date, both pathogenic and protective effects of γδ T cells on pregnancy have been reported. Psarra et al. found that a significantly higher number of RSA patients harbored an increased population of γδ T cells in peripheral blood (16), Talukdar et al. found that IFNγ- and IL-17-producing γδ T cells were increased in RSA patients (17), and both groups suggested that these γδ T cells are pathogenic. On the other hand, human decidual γδ T cells showed a dominantly TGF-β- and IL-10-expressing profile during early pregnancy (18), and later investigation reported that these decidual γδ T cells could upregulate the biological functions of trophoblasts *via* IL-10 secretion in early human pregnancy (19) and thus ensure the success of pregnancy.

Human γδ T cells are a heterogeneous population that can be divided into subpopulations according to their TCR usage (e.g., Vδ1, Vδ2, and Vδ3). Vδ2^+^ cells are the predominant γδ T-cell subsets in the peripheral blood, and these cells are often regarded as an innate-like cells by carrying a semi-invariant Vγ9(JP)Vδ2 TCR. Most Vδ2^-^ γδ T cells are Vδ1^+^ cells that distribute in tissues and exhibit adaptive-like features (7). Each subpopulation is further subject to the regulation of local microenvironment and antigen stimuli and then differentiates into diverse functional populations, which is similar to Th1, Th2, Th17, and Treg cells, or expresses a different array of molecules related to cytotoxicity (e.g., perforin, granzyme B, Fas ligand, and CD107a) (20) and activation/exhaustion (e.g., CD25, PD1 and TIM-3) (21). Therefore, the distinct results described above could be due to the functional heterogeneity of γδ T-cell subsets. To reconcile this discrepancy, this study was designed to characterize the subpopulations and functional profiles of peripheral γδ T cells from RSA patients and healthy women with or without pregnancy by flow cytometry. In addition, the decidua from pregnant women with or without RSA was stained for γδ T cells by immunohistochemistry (IHC) and bulk sequenced for gene expression to compare the functional properties of local γδ T cells.

## Method

### Study Participants

Twenty-one RSA patients, namely, 10 pregnant (RSA-P) women and 11 un-pregnant (RSA-UP) women, were included for this study. RSA patients were defined as women with a history of two or more consecutively miscarriage without genetic, endocrine, uterine, or autoimmune abnormalities as well as other infections. Twenty-one age-matched healthy women with no history of miscarriage and without other complications or infections, namely, 11 healthy pregnant (H-P) and 10 healthy un-pregnant (H-UP) women, were recruited as controls for comparison. Of note, subjects who had a positive test result for hepatitis B surface antigen (HBsAg), anti-cardiolipin antibody (ACL), Beta-2 glycoprotein 1 antibodies (β2GP1 Ab), lupus anticoagulant (LAC), or thyroglobulin (Tg) were also excluded from this study to make the comparison results interpretable. Clinical characteristics of the participants are presented in **Table 1**. This study was approved by the institute ethics committee of Tongji Hospital (Ref. No. TJ-C20180201), and the informed consents were obtained from all participants.

**Table 1:** Clinical characteristics of the participants in this study.

	RSA pregnant RSA-P (*n* = 10)	Healthy pregnant H-P (*n* = 11)	RSA un-pregnant RSA-UP (*n* = 11)	Healthy un-pregnant H-UP (*n* = 10)
**Age (years)**	31.2 ± 4.8	31.1 ± 3.9	30.8 ± 3.1	26.5 ± 3.8
**Gravidity**	3.5 ± 1.6	2.7 ± 1.0	2.5 ± 0.7	0.2 ± 0.6
**Parity**	0.1 ± 0.3	1.3 ± 0.5	NA	0.2 ± 0.6
**Sp. Abortions**	2.9 ± 0.7	NA	2.3 ± 0.5	NA
**Gestational weeks**	8.4 ± 1.8	7.5 ± 0.9	NA	NA

Data were expressed as mean ± SD.

### PBMC Isolation

Peripheral blood samples from the four groups were collected in heparin anti-coagulated vacutainer tubes (BD Pharmingen, CA, USA). After diluted twice by PBS, the blood was added carefully onto the Ficoll layer (Blood: PBS: Ficoll = 1:1:1) and centrifuged at 800 × *g* for 30 min with brake off. Mononuclear layer was obtained and washed twice by PBS. The PBMC aliquots were used immediately for Flow Cytometry Staining or stocked frozen in fetal calf serum containing 10% DMSO and 5% Dextran at −80°C until further test.

### Cell Labeling and Flow Cytometric Analysis

For surface staining, freshly collected blood was aliquoted (100 μl) and stained by fluorochrome-conjugated monoclonal antibodies against CD3, γδ TCR, Vδ1, Vδ2, and PD1. After incubating with the mAbs for 30 min at 4°C in the dark, the blood sample was lysed by 2 ml of lysis buffer (BD Pharmingen) for 8 min, washed twice by 2 ml of ice-cold FACS buffer (PBS containing 2% FCS and 0.1% azide), and then fixed in 300 μl of 1% paraformaldehyde for flow cytometric analysis.

For intracellular staining of cytotoxicity-related molecules, 3 × 10^5^ fresh isolated PBMCs were stained by fluorochrome-conjugated monoclonal antibodies against Vδ1, Vδ2, and CD107a for 30 min at 4°C in the dark and then stained for intracellular perforin and granzyme B after fixation and permeabilization by the Cytofix/Cytoperm Kit (BD Pharmingen). The cells were washed twice before resuspended in 300 μl of 1% paraformaldehyde for flow cytometric analysis.

While for intracellular cytokine staining, 1 × 10^6^ fresh PBMCs were stimulated by a cocktail containing phorbol myristate acetate (PMA), ionomycin, and brefeldin a (BD Pharmingen) for 5 h in 24-well flat-bottom plates, thereafter, the cells were harvested and washed once by PBS for immunolabeling. The cells were stained by the abovementioned fluorochrome-conjugated monoclonal antibodies against Vδ1 and Vδ2 for 30 min at 4°C in the dark and then divided into two parts and stained for IFNγ and IL-4, or TNFα and IL-17A, respectively, after fixation and permeabilization. Finally, the cells were washed twice and resuspended in 300 μl of 1% paraformaldehyde for flow cytometric analysis.

All the fluorochrome-conjugated monoclonal antibodies were listed in **Supplementary Table S1**. The flow cytometric analysis was performed on an BD LSR Fortessa instrument (BD Bioscience) and data were analyzed by FlowJo V10 (BD Bioscience).

### Tissue Preparation

Decidua tissues were all collected from pregnant participants (the RSA-P and H-P subjects). After washing twice by PBS, the tissues were cryopreserved immediately for further RNA sequencing and qRT-PCR, or fixed with 4% paraformaldehyde for 48 h, and then embedded in paraffin wax and sectioned at 3 µm. The paraffin sections were proceeded and stained with hematoxylin/eosin for histological investigation; selected slides were used for IHC (2.7) and multicolor IHC (mIHC, 2.8).

### RNA Sequencing and Data Analysis

The sample information for bulk sequencing is listed in **Table 2**. Total RNAs of decidua tissues were extracted using Trizol (Invitrogen, CA, USA) according to the manufacturer’s instruction. Oligo(dT)-attached magnetic beads were used to purify mRNA, which then was fragmented into small pieces with fragment buffer at the appropriate temperature to generate the final library. The final library was amplified with phi29 to make DNA nanoball (DNB), which has more than 300 copies of one molecule; DNBs were loaded into the patterned nanoarray, and single-end 50-base reads were generated on the BGIseq500 platform (BGI-Shenzhen, China).

**Table 2:** Clinical characteristics of the participants recruited for RNA-seq.

Group	Sample	Age (years)	Gravidity	Parity	Spontaneous Abortion	Artificial Abortions	Sample GA/(D)
RSA-P	RSA01	30	4	0	4	0	62
	RSA02	31	2	0	2	0	57
	RSA03	34	3	0	3	0	56
HC-P	HC01	33	3	2	0	1	55
	HC02	33	3	1	0	2	61
	HC03	23	1	0	0	1	54

For differentially expressed gene (DEG) analysis, RNA-seq data were mapped to human genome (GRCh38) using HISAT2 (v2.2.0). FeatureCounts integrated into Subread (v2.0.0) was used for counting reads with GENECODE gene annotation (v34). Then, the analysis of DEGs was performed using DESeq2 R package (v1.30.0). DEGs were selected as follows: adjusted *p*-value < 0.05 and |log2FoldChange| > 1. Gene counts were normalized by DESeq2 and converted to log_2_(normalized counts +1) format. With DESeq2, the Wald test is the default used for hypothesis testing of DEG analysis. The Benjamini and Hochberg method was used to adjust *p*-value. Log_2_FoldChange was calculated as follows: log_2_(normalized counts group 1/normalized counts group 2). *Z*-score of normalized gene expression by genes (each gene with six samples has a zero mean and standard deviation is 1) was shown by hierarchical clustering heatmap.

For GO and KEGG enrichment analysis, all DEGs found above were used for enrichment analysis. ClusterProfiler R package (v3.18.0) was used to perform GO (including Biological Process, Cellular Component and Molecular Function) and KEGG enrichment analysis. *p*-value was calculated based on hypergeometric test. The Benjamini and Hochberg method was used to adjust *p*-value. Significantly enriched GO term and KEGG pathway were selected under the following criteria: adjusted *p*-value < 0.05.

The abundances of immune cells were estimated by CIBERSORTx web server and the LM22 signature matrix, which contains 547 genes distinguishing 22 human hematopoietic cell phenotypes. TPM was used for normalizing gene expression levels. B-mode batch correction was enabled and quantile normalization was disabled.

BCR and TCR clonotypes were recovered from RNA-seq data using Mixcr (v.3.0.12). “Analyze Shotgun”, a single command integrating a complicated execution pipelines for RNA-seq data, was used with parameters “impute-germline-on-export” and “only-productive”. Unique clonotype was defined as BCR/TCR with specific CDR3 nucleotide sequence, V/J segments, and hypermutations.

### qRT-PCR

Total RNAs of decidua tissues were extracted using Trizol (Invitrogen) according to manual instructions. Quantitative RT-PCR (qRT-PCR) was performed using the HiScriptII Supermix (Vazyme, Nanjing, China) following the manufacturer’s instructions. Briefly, RNA was quantified using a Nanodrop One instrument (Thermo, MA, USA) and 1 μg was used for reverse transcription using random primers. For qRT-PCR, SYBR Green master mix and primers (final concentration at 200 nM) were used and results were analyzed in CFX Connect PCR detection system (Bio-Rad, CA, USA). Primers were designed according to a previous publication (22) for *CCL8* and *GAPDH*, or using an open resource (www.ncbi.nlm.nih.gov/tools/primer-blast) for *TRDV1*, *TRDV2*, and *TRDV3*, whose sequences are listed in **Supplementary Table S2**. Expression levels were normalized to *GAPDH* and represented as fold change compared to the control (2^−ΔΔCt^).

### Immunohistochemistry

Paraffin sections were dewaxed by dimethylbenzene and rehydrated by gradually reduced concentration of ethanol. Then, the slides were boiled in AR6 (antigen retrieval solution) for 15 s and incubated for 15 min, followed by cooling in ice water for 20 min. Endogenous peroxidase activity was blocked with 4% hydrogen peroxide for 15 min at room temperature, and non-specific binding was blocked with 1% bovine serum albumin/PBS (1% BSA) solution for 1 h; 1:150 diluted primary mouse anti-human TCRγδ mAb (H-41, Abcam) in 1% BSA was used for overnight incubation at 4°C; 1:200 diluted HRP-conjugated goat anti-mouse IgG mAb (Akoya Biosciences) in 1% BSA was used as secondary antibody for 1 h incubation at 37°C. The HRP activity was revealed with ready-to-use 3,3-diaminobenzidine tetrahydrochloride (DAB) for 3 min, and nuclei was slightly counterstained with hematoxylin. The slides were always washed three times between each step with 1‰ Tween20 and 0.3‰ Triton X-100 in PBS. After staining, samples were dehydrated by gradually increased concentration of ethanol. Finally, the slides were mounted with resinene for microscope observation. The images were obtained by DSY2000X (UOP, Chongqing, China).

### Multicolor IHC (mIHC)

mIHC was performed using Opal 7-Color Manual IHC Kit (Akoya Biosciences, DE, USA) following the manufacturer’s instructions. Briefly, dewaxed and rehydrated sections were antigen retrieved and blocked for endogenous peroxidase and Fc receptor using the same procedure as described in the *Immunohistochemistry* section. The samples were incubated in mouse anti-human TCRγδ mAb (H-41, Abcam, 1:150 diluted) for 1 hour at 37°C, HRP-conjugated goat anti-mouse IgG mAb (1:200 diluted) for 30 min at 37°C, Opal520 fluorescein (1:100 diluted in amplification buffer) for 10 min at 37°C, successively. The nonspecific staining was removed by repeating the procedure of antigen-repair through AR6. The samples were then incubated with mouse anti-human CD107a mAb (H4A3, BD, 1:100 diluted) for 1 h at 37°C, HRP-conjugated goat anti-mouse IgG mAb (1:200 diluted) for 30 min at 37°C, and Opal620 fluorescein (1:100 diluted in amplification buffer) for 10 min at 37°C, successively. The slides were always washed three times between each step with 1‰ Tween20 and 0.3‰ Triton X-100 in PBS. After staining, samples were stained for cellular DNA with DAPI and mounted with Fluoromount-G (SouthernBiotech, AL, USA). The images were obtained by DSY2000X (UOP, Chongqing, China). The cells with green fluorescence were counted, in which red fluorescence was checked for each cell, and finally the frequency of CD107a-expressing cells in decidual γδ T cells was assessed in a blinded manner by two independent pathologists.

### Statistical Analysis

Statistical analyses were performed with GraphPad Prism software (Version 6.0, GraphPad Software Inc.). Throughout the study, *n* refers to the number of subjects where every subject is one data point. Unpaired two-group comparisons were done with Mann–Whitney *U*-test. Paired two-group comparisons were done with Wilcoxon matched-pairs signed rank test. In figures, ***p < 0.001, **p < 0.01, and *p < 0.05. In tables, continuous variables were described as mean ± SD and compared using two-tailed Student’s *t*-test or Mann–Whitney *U*-test for two groups depending on distribution.

## Results

### Unchanged Frequency of γδ T Cells but Increased PD1 Expression on γδ T Cells in Pregnant RSA Patients

**Figure 1:**
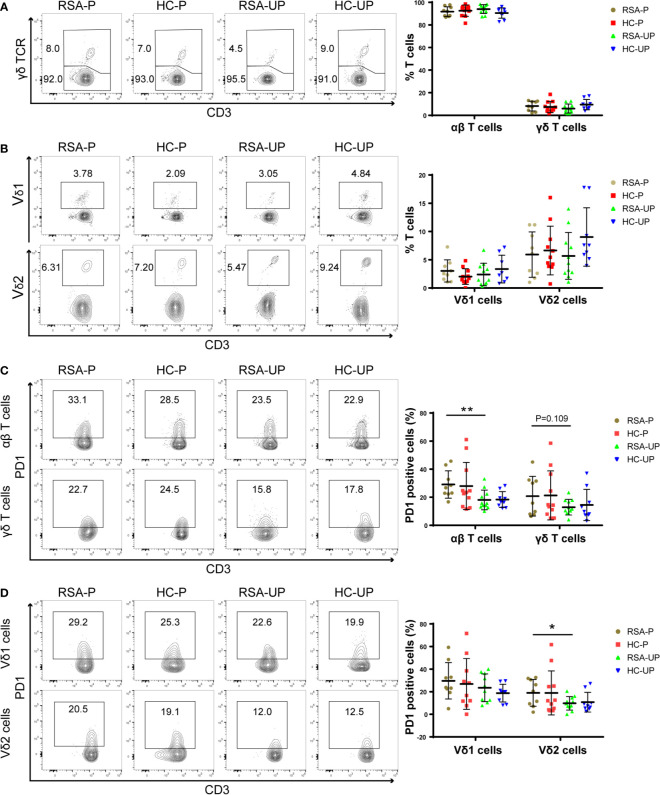
The frequencies of T-cell subsets and their PD1 expression in the peripheral blood of RSA patients and healthy controls with or without pregnancy. Peripheral blood samples from four groups were stained for T-cell subsets and PD1 expression. **(A)** The frequencies of αβ T cells and γδ T cells in all T cells. **(B)** The frequencies of Vδ1^+^ cells and Vδ2^+^ cells in total T cells. **(C)** PD1 expression on αβ T cells and γδ T cells. **(D)** PD1 expression on Vδ1^+^ cells and Vδ2^+^ cells gated from total T cells. Left, representative flow cytometry plots; right, statistical data show mean ± s.e.m. Statistical analyses using Mann–Whitney *U*-test for the same cells among different groups. Differences are indicated: **p < 0.01 and *p < 0.05. RSA-P, RSA pregnant, *n* = 10; HC-P, healthy pregnant, *n* = 11; RSA-UP, RSA unpregnant, *n* = 11; HC-UP, healthy unpregnant, *n* = 10.

Previous reports have shown that the frequency of γδ T cells changed during pregnancy in the peripheral blood of RSA patients (16, 17, 23–27), while other studies did not support this notion (28, 29). We therefore sought to examine this issue in our cohort (see *Study Participants* section). Peripheral blood samples were stained for γδ T cells by flow cytometry, and the gating strategy was shown (**Supplementary Figure S1A**). The frequencies of αβ T and γδ T cells in all T cells were comparable between all four groups (**Figure 1A**), so were the frequencies of γδ T subsets (i.e., Vδ1^+^ and Vδ2^+^ cells) in all T cells (**Figure 1B**) or in γδ T cells (**Supplementary Figure S1B**). When the ratios of different γδ T-cell subsets were compared, the ratio of Vδ2^+^ to Vδ1^+^ cells tended to decrease in RSA patients, which did not reach significant difference (**Supplementary Figures S1C, D**). These results indicated the unchanged frequency of γδ T cells among different groups in our cohort.

We further examined T cells and their subsets for the expression of PD1, a molecule mainly expressed on activated T cells and was frequently used as an activation and exhaustion marker for immune cells (30). We found an increased PD1 expression on αβ T cells in the RSA-P group compared to the RSA-UP group, whereas PD1 expression on γδ T cells were not significantly different between these two groups, despite the fact that we did notice an increased tendency (*p* = 0.109, **Figure 1C**). We went on to determine the expression of PD1 on γδ T-cell subsets and found that the frequency of PD1^+^Vδ2^+^ cells was increased in the RSA-P group compared to the RSA-UP group (**Figure 1D** and **Supplementary Figure S1E**).

Taken together, our results indicated that the frequency of γδ T cells was unchanged in RSA patients compared to healthy controls and was relatively stable during physiological and pathological pregnancy. However, PD1 expression on Vδ2^+^ cells and αβ T cells was significantly increased in RSA patients during pregnancy (RSA-P *vs*. RSA-UP). Thus, the delicate control of PD1 expression on these two cell populations may be required for successful pregnancy.

### Increased Cytotoxicity of γδ T Cells in the RSA-P Group

**Figure 2:**
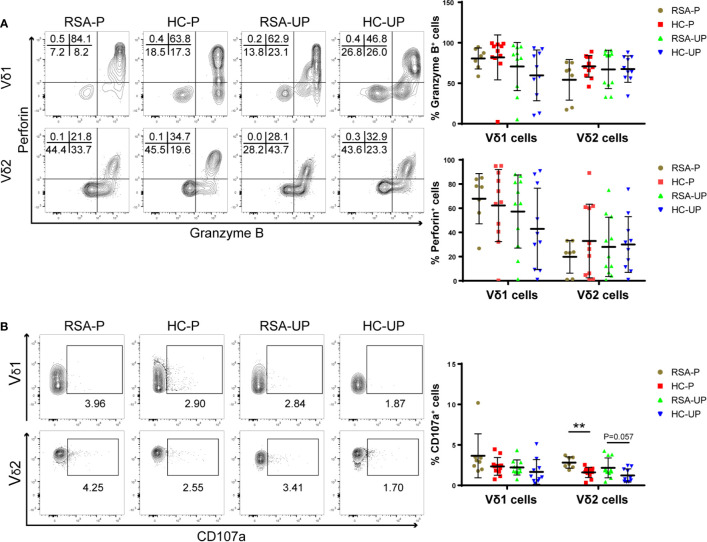
The frequencies of perforin^+^, GZMB^+^, and CD107a^+^ cells in peripheral γδ T cells in RSA patients and healthy controls with or without pregnancy. PBMCs were obtained from peripheral blood of each subject and directly stained for membrane CD107a as well as intracellular perforin and GZMB. **(A)** The frequencies of GZMB^+^ and perforin^+^ cells in Vδ1^+^ cells and Vδ2^+^ cells from four groups. **(B)** The frequencies of CD107a^+^ cells in Vδ1^+^ cells and Vδ2^+^ cells from four groups. Left, representative flow cytometry plots; right, statistical data show mean ± s.e.m. Statistical analyses using Mann–Whitney *U*-test among different groups. Differences are indicated: **p < 0.01. RSA-P, RSA pregnant, *n* = 10; HC-P, healthy pregnant, *n* = 11; RSA-UP, RSA unpregnant, *n* = 11; HC-UP, healthy unpregnant, *n* = 10.

It was reported that decidual γδ T cells in early pregnancy expressed cytotoxic molecules such as perforin, granzyme A (GZMA), granzyme B (GZMB), FasL, and Granulysin (20), which were potentially detrimental to the fetus; we therefore explored cytotoxicity-related phenotypes of γδ T cells to reveal the functional state of γδ T cells during spontaneous abortion. For this purpose, PBMCs were obtained from the blood and stained for the expression of GZMB, perforin, and CD107a on γδ T cells/subsets. The gating strategy is shown in **Supplementary Figure S2A**. There was no significant difference between four groups in terms of the frequencies of GZMB^+^ (**Figure 2A**), perforin^+^ (**Figure 2A**), and GZMB^+^perforin^+^ double-positive cells (**Supplementary Figure S2B**) in both Vδ1 and Vδ2 cells.

Interestingly, we observed an increased tendency regarding the frequency of CD107a^+^ cells in Vδ2 cells in un-pregnant RSA patients compared to un-pregnant controls (RSA-UP *vs*. H-UP, *p* = 0.0565, **Figure 2B**), and the difference was more evident in the RSA-P *vs*. H-P comparison (*p* < 0.01, **Figure 2B**). CD107a is a molecule exported to the surface of cytotoxic cells upon cell degranulation (31); therefore, increased expression of CD107a can reflect the elevated killing capability of γδ T cells. Together, our results indicated that even without the increased expression of GZMB and perforin, γδ T cells in RSA patients might still have elevated cytotoxicity by increasing their CD107a expression and contribute to the recurrent spontaneous abortion.

### Increased IL-17A-Secreting Vδ2^+^ Cells but Not IL-4-Secreting Vδ2^+^ Cells in Peripheral Blood From RSA Patients During Pregnancy

**Figure 3:**
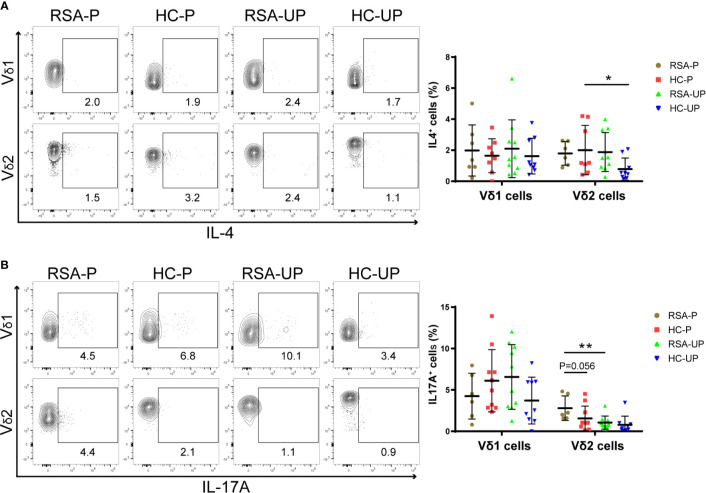
The cytokine profiles of Vδ1^+^ and Vδ2^+^ γδ T cells in the peripheral blood of RSA patients and healthy controls with or without pregnancy. PBMCs were obtained from peripheral blood of each subject and stimulated with PMA/Ionomycin/Brefeldin A for 5 h before being stained for intracellular cytokines. **(A)** The frequencies of IL-4-secreting cells in Vδ1^+^ cells and Vδ2^+^ cells from four groups. **(B)** The frequencies of IL-17A-secreting cells in Vδ1^+^ cells and Vδ2^+^ cells from four groups. Left, representative flow cytometry plots; right, statistical data show mean ± s.e.m. Statistical analyses using Mann–Whitney *U*-test among different groups. Differences are indicated: **p < 0.01, and *p < 0.05. RSA-P, RSA pregnant, *n* = 10; HC-P, healthy pregnant, *n* = 11; RSA-UP, RSA unpregnant, *n* = 11; HC-UP, healthy unpregnant, *n* = 10.

Both decidual and peripheral γδ T cells have been reported to exhibit Th1-, Th2-, and Th17-like phenotypes, the balance of which during gestation has been suggested to be related to the outcome of pregnancy (18, 26, 32, 33). Therefore, we next set out to examine the balance of these cytokine-defined γδ T-cell subsets in RSA patients and healthy controls. PBMCs were obtained from the blood and stimulated with PMA/Ionomycin/Brefeldin A for 5 h before being stained with antibodies against IFNγ and IL-4 or TNFα and IL-17A, and the gating strategy is shown in **Supplementary Figure S3A** using a representative sample. There was no significant difference regarding the frequency of IFNγ^+^ and TNFα^+^ cells in Vδ1^+^ and Vδ2^+^ γδ T cells among four different groups (**Supplementary Figures S3B, C**). However, the IL-4-secreting Vδ2^+^ cells were significantly increased in pregnant healthy controls compared to un-pregnant controls (HC-P *vs*. HC-UP), whereas the same cells were not different in the comparison between the RSA-P and RSA-UP groups (**Figure 3A**). On the contrary, increased IL-17A-secreting Vδ2^+^ cells were only evident in the RSA-P *vs*. RSA-UP comparison, but not in the HC-P *vs*. HC-UP comparison (**Figure 3B**). Interestingly, the IL-17A-secreting Vδ2^+^ cells also tended to be increased in the RSA-P group compared to the HC-P group (**Figure 3B**). Together, our results suggested that the upregulation of IL-4-secreting Vδ2^+^ cells and the relatively stable frequency of IL-17A-secreting Vδ2^+^ cells may be required for maintaining healthy pregnancy, whereas the dysregulation of these cells could contribute to the occurrence of RSA.

### Inflammatory Microenvironment in the Decidua of RSA Patients

**Figure 4:**
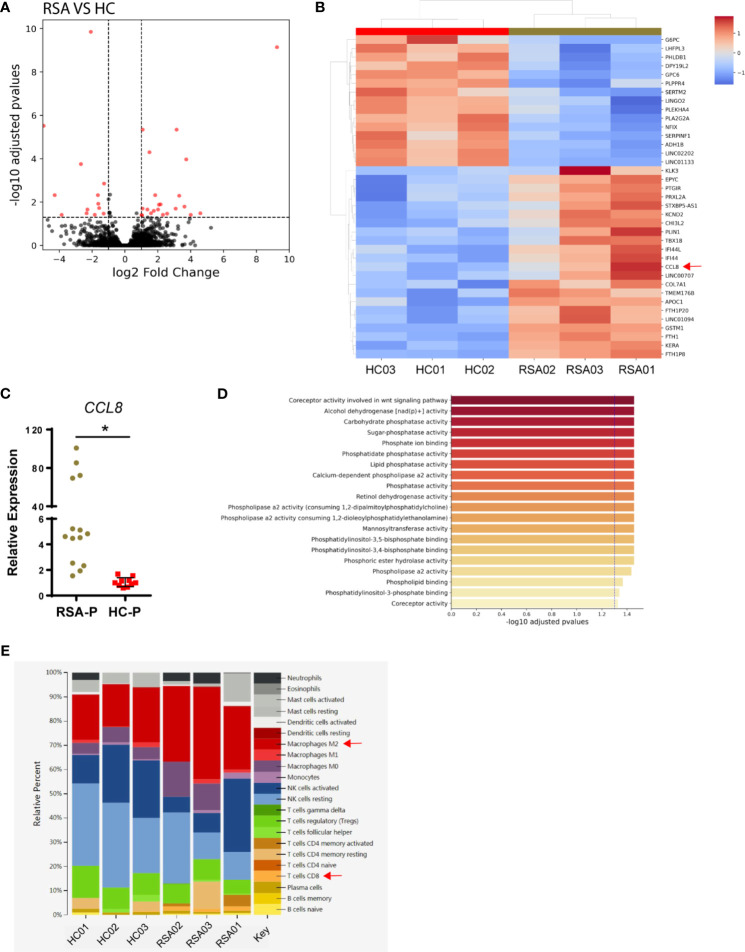
Transcriptomic analysis of decidual samples from RSA-P patients and HC-P subjects. Bulk RNA-seq was performed with decidual samples from RSA-P patients (*n* = 3) and HC-P subjects (*n* = 3). **(A)** Volcano plots illustrating differentially expressed genes (DEGs) between RSA-P patients and HC-P controls. **(B)** Heatmap showing DEGs between RSA-P and HC-P patients. **(C)** qRT-PCR analysis for *CCL8* expression in RSA-P and HC-P decidua with 14 samples in each group. **(D)** GO analysis for the DEGs. **(E)** CIBERSORT analysis for the immune cells. Statistical data show mean ± s.e.m. Statistical analyses using Mann–Whitney *U*-test among different groups. Differences are indicated: *p < 0.05.

To explore the changes of local microenvironment in the feto-maternal interface during early pregnancy in RSA, we next examined the transcriptional profiles of the decidua tissue by RNA sequencing using samples from three RSA patients and three healthy controls (**Table 2**). Principal component analysis was used to reveal the overall gene expression pattern for each sample (**Supplementary Figure S4A**). Differential gene expression analysis for the RSA-P and HC-P groups found that 44 genes were differentially expressed (**Figures 4A, B**), of which the *CCL8* gene was upregulated in the RSA-P group. The increased *CCL8* expression in the RSA-P group was further confirmed by qRT-PCR using more samples (**Figure 4C**), suggesting the potential proinflammatory role of CCL8 molecule in the feto-maternal interface during pathological pregnancy by recruiting immune cells. Of note, the differently expressed genes were mainly enriched into the glycolysis pathway (**Figure 4D** and **Supplementary Figure S4B**), which might be caused by the majority of stroma cells in the decidua, as determined by hematoxylin–eosin staining (data not shown). Since the upregulated *CCL8* could result in the increased recruitment of multiple types of immune cells to exaggerate the inflammatory conditions, the distribution of immune cells was further analyzed by the CIBERSORT algorithm. This analysis demonstrated that CD8^+^ T cells and M2 macrophages were increasingly distributed in the RSA-P group (**Figure 4E** and **Supplementary Figure S4C**).

In sum, our transcriptomic analysis indicated that the upregulated expression of *CCL8* increased infiltration of CD8^+^ T cells and M2 macrophages, and more vigorous glycolysis may orchestrate to promote an inflammatory microenvironment in the feto-maternal interface in RSA patients.

### Higher CD107a Expression on Decidual γδ T Cells in RSA Patients

**Figure 5:**
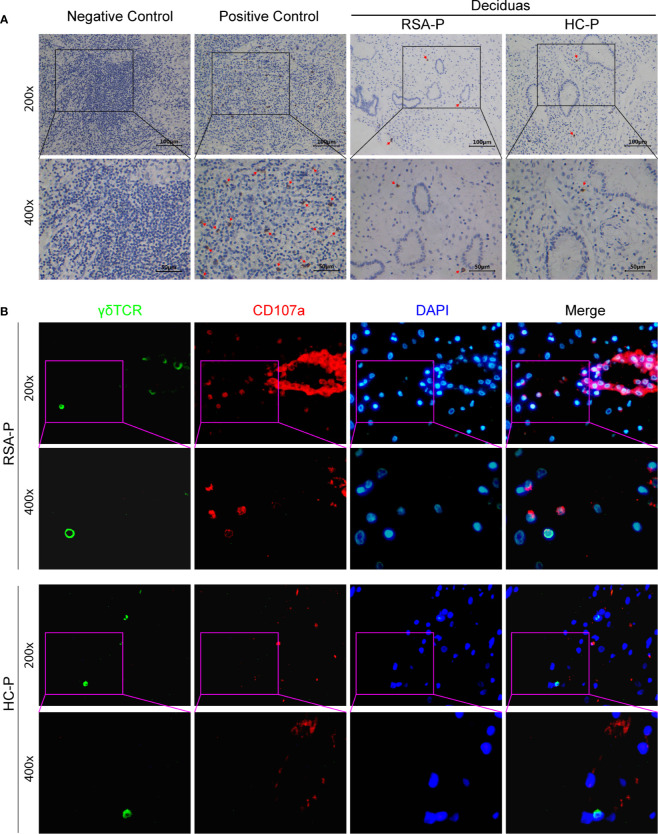
The presence of CD107a^+^γδ T cells in the decidua of pregnant RSA patients. **(A)** Representative IHC staining results for γδ T cells in decidua samples from an RSA-P patient and a H-P control (right); tumor samples were used as negative and positive control. Positive staining was indicated by the red arrow. **(B)** Representative mIHC staining results of γδ TCR, CD107a, and DAPI with decidua samples from an RSA-P patient and a H-P control. γδ TCR was labeled with Opal 520 (green), CD107a was labeled with Opal 620 (red), and the nuclei were stained with DAPI (blue).

Since γδ T cells could either secrete CCL8 (34, 35) or respond to CCL8 by expressing its receptors such as CCR1 (36), CCR2 (36–38), CCR3 (39), and CCR5 (40), we therefore examined the decidual presence of γδ T cells in the RSA-P and HC-P groups by IHC. γδ T cells can be found in the decidua of both RSA-P and HC-P women, but there was no significant difference regarding the proportion of γδ T cells between these two groups, and the infiltration of this cell was relatively low when using tumor samples as positive control (**Figure 5A**). We also recovered the sequence information of BCR and TCR chains from our RNA-seq data; no difference was found in terms of the numbers of recovered TRD and TRG chains, which reflect the diversity of TCR-γ and -δ chains, respectively (**Figure S5A**). TRD was further analyzed by qRT-PCR using more samples, and no difference was found as well (**Figure S5B**). Next, to determine if the expression of the cytotoxic molecule CD107a is also upregulated in the decidua of RSA-P patients, especially on γδ T cells, as indicated by our previous finding in peripheral blood, we stained for the expression of CD107a and γδ T cells simultaneously using multicolor IHC. Our result showed a higher expression of CD107a in RSA-P patients compared to HC-P controls (**Figure 5B** and **Supplementary Figure S5C**), and the co-localization of CD107a and γδ T cells can be found in RSA-P patients but rarely in healthy controls. Together, although decidual γδ T cells had similar diversity in RSA-P and HC-P women, these cells may increase their CD107a expression during RSA and thus contribute to spontaneous abortion.

## Discussion

Previous studies have reported the increased γδ T cells in the peripheral blood during normal pregnancy (17, 23, 24, 27), suggesting that these cells are important for successful pregnancy (24, 25). However, no consistent conclusion has been reached regarding the alteration of γδ T cells in RSA patients compared to healthy controls, with some investigations showing the increase of these cells in RSA (16, 41), whereas others provide data that do not support this notion (28, 42). This discrepancy disappeared when examining γδ T cell subsets, since the increased frequency of Vδ2+ cells, which is the predominant subsets in peripheral γδ T cells, was unanimously observed in RSA patients (43). Therefore, we examined these issues in our cohort, which included 42 RSA patients and healthy controls with or without pregnancy (see *Study Participants* section). We found that the frequency of γδ T cells and the Vδ1/Vδ2 ratio were comparable among the four different groups. Thus, our results do not support the notion that the frequencies of peripheral γδ T cells and their subsets are significantly changed during early pregnancy in either RSA patients and healthy controls. However, further investigations including more subjects are required to confirm these results. One interpretation for this discrepancy might be the dynamic distribution of γδ T cells during pregnancy (43, 44). Alternatively, it could be the phenotype but not the number of γδ T cells/subsets that is actually altered in RSA.

To investigate the phenotypic changes of peripheral γδ T cells and their subsets in RSA, we next examined the PD1 expression on maternal circulating γδ T cells/subsets and other T cells and found that elevated PD1 expression was evident on Vδ2^+^ cells and αβ T cells in the RSA-P group compared to that in the RSA-UP group. However, the increased PD1 expression on these cells was not observed in the HC-P *vs*. HC-UP comparison or in the RSA-P *vs*. HC-P comparison like others (42), suggesting that Vδ2^+^ cells and αβ T cells might upregulate their PD1 expression during pathological pregnancy. PD1 is a molecule expressed upon T-cell activation, which then acts as a checkpoint to prevent exaggerated T-cell activation through interacting with its ligand, PD-L1 or PD-L2 (45). In the past few decades, PD1-expressing T cells have been well-established as an exhausted T-cell population with defective function generated during chronic viral infection and tumor development (46, 47). Accordingly, targeting these cells by PD1 blockade has resulted in great clinical success in cancer immunotherapy for a variety of cancer types (48). Not surprisingly, PD1^+^ αβ T cells and γδ T cells were also identified during pregnancy and were often suggested as exhausted T cells that are protective for normal pregnancy (49). However, PD1^+^ T cells may contain not only exhausted T cells, but also non-exhausted T cells that are activated. An early study reported that upon stimulation with viral antigen, PD1^+^ T cells exhibited polyfunctionality, as indicated by their capacity to produce TNFα and IFNγ and express CD107a (50). PD1 expression was also frequently used as an activation marker for T cells in other investigations (50–52). Therefore, PD1^+^ Vδ2^+^ cells and αβ T cells could simply be activated T cells with proinflammatory function, and their increase in RSA may suggest the pathogenicity of these cells in RSA. Nevertheless, further studies are warranted for examining the functionality of these PD1^+^ T cells, not only in the periphery blood but also in the decidua, to offer a precise understanding of the role of these cells in physiological and pathological pregnancy.

Vδ2^+^ cells are the major subpopulation of γδ T cells in the periphery. It has been reported that these γδ T cells are able to utilize perforin-GZMB, Fas-FasL, and TRAIL pathways to kill target cells upon activation (53). The upregulation of PD1 on these cells in RSA prompted us to test whether they also have altered cytotoxicity. For this purpose, we compared the expression of cytotoxicity-related molecules (i.e., perforin, GZMB, and CD107a) on periphery Vδ2^+^ cells from RSA patients and health controls with or without pregnancy. We found that increased CD107a expression was evident on Vδ2^+^ cells in RSA-P patients in comparison to that in H-P subjects. Interestingly, the frequency of the same cell also showed an increased tendency in un-pregnant RSA patients compared to un-pregnant controls. Together, these results suggest that cytotoxic CD107a^+^Vδ2^+^ cells could be pathogenic in RSA, and further increased frequency of these cells may lead to the failure of pregnancy. However, we failed to find any difference in all four groups compared in terms of the expression of perforin and GZMB on Vδ2^+^ cells and Vδ1^+^ cells. The differential expression patterns of the above two molecules and CD107a could be due to the different detection methods used. For perforin and GZMB, the two main cytotoxic molecules that are stored in the intracellular granules of cytotoxic cells (54) and are released upon stimulation (55), intracellular staining was used in this study according to the protocol used by numerous investigations (54), the result of which can only reflect the cytotoxic potential of cytotoxic cells. However, for CD107a, a protein constitutively expressed on the lysosomal membrane and exported to the cell membrane upon stimulation, we used direct surface staining for *ex vivo* cells without any *in vitro* stimulation (56–58); thus, our *ex vivo* measurement can faithfully recapitulate the *in vivo* expression pattern of CD107a. Of note, we did not find any change of CD107a expression on peripheral γδ T cells during normal pregnancy (i.e., HP *vs*. H-UP, **Figure 2B**), which is different from a recent study by Norenberg et al. (59). Using an *in vitro* stimulation protocol with ionomycin and PMA, they found that CD107a expression on peripheral γδ T cells was increased on CD56^-^ γδ T cells in the first trimester and decreased slowly in the second as well as the third trimester. Given the low proportion of CD56^+^ γδ T cells and the high frequency of Vδ2^+^ cells among peripheral γδ T cells, the CD107a expression on total Vδ2^+^ cells was very likely increased as well during healthy pregnancy in their study. The distinct results by this investigation and ours could again be attributed to the aforementioned different methods used for detecting the surface expression of CD107a. Interestingly, Norenberg et al. also determined the co-expression of CD107a and PD1 on γδ T cells and argued that PD1 expression could represent a negative feedback mechanism used by cytotoxic CD107a^+^ γδ T cells to downregulate their cytotoxicity and secure the success of pregnancy. Nevertheless, the co-expression of CD107a and PD1 on γδ T cells should be measured in the future investigation to test this hypothesis.

Distinct from peripheral blood, the feto-maternal interface (i.e., the decidua) harbors a very unique immune milieu (60). Furthermore, CD107a expression was reported to be lower in decidua γδ T cells than that in circulating γδ T cells (61); therefore, we then interrogate if CD107a is similarly overexpressed on decidua γδ T cells as on peripheral γδ T cells in RSA. As revealed by IHC, γδ T cells can be found in the decidua of both RSA-P patients and H-P controls at similar frequency (**Figure 5A**). However, when γδ T cells and CD107a were simultaneously stained by mIHC, CD107a-expressing γδ T cells could be readily detected in RSA-P patients but rarely in H-P controls (**Figure 5B**). Of note, CD107a-expressing non-γδ T cells were also more frequently observed in RSA-P patients than in H-P controls (**Figure 5B**). Collectively, our results indicated that CD107a-expressing γδ T cells in both periphery and decidua might be pathogenic in RSA, and other cytotoxic cells may also contribute.

Historically, Th1/Th2 imbalance has been frequently used to explain the immune puzzle of pregnancy, during which maternal immune system is inevitably aware of semi-allogenic fetal antigens without rejecting the conceptus (62). According to this paradigm, maternal immune system should be adapted to be Th2 biased to prevent allorejection of the conceptus. In parallel to αβ T cells, γδ T cells can also be classified into Th1-, Th2-, Th17-, and Treg-like subsets according to their distinct cytokine profiles (63–66). Therefore, it is not totally surprising that the same paradigm was also applied in interpreting the role of γδ T-cell subsets in physiological and pathological pregnancy. For example, various studies have reported the Th2 bias in the decidual γδ T cells characterized by secreting TGFβ and IL-10 (18), suggestive of their importance in maintaining successful pregnancy. On the contrary, Th1- and Th17-like γδ T cells were found to be higher in RSA patients than that in healthy controls (17, 67, 68), and so was the Th1/Th2 ratio (69), indicating the pathogenic role of Th1- and Th17-like γδ T cells in RSA. In the present study, higher frequency of IL-4-secreting Vδ2 cells was only found in the HC-P *vs*. H-UP comparison, but not the RSA-P *vs*. RSA-UP comparison. This difference in IL-4-secreting Vδ2 cells was in stark contrast to that in IL-17A-secreting Vδ2 cells, the upregulation of which was only observed in the RSA-P *vs*. RSA-UP comparison, but not in the HC-P *vs*. H-UP comparison. Thus, our results are in agreement with previous publications in suggesting the pathogenic role of IL-17A-secreting γδ T cells and the protective role of IL-4-secreting γδ T cells during pregnancy (70–72), and further specify that these cells belong to the Vδ2 compartment. Of note, we did not find any difference between groups regarding the frequencies of TNFα- and IFNγ-secreting Vδ2 cells, which were reported as Th1-like and suggested to be pathogenic in RSA (69). The reason could be that the frequencies of these cells are already very high (with the median of 89.31% and 94.72% for TNFα- and IFNγ-secreting Vδ2 cells, respectively) (**Supplementary Figures S3B, C**) in un-pregnant healthy controls from our cohort; thus, further increase is very difficult to be observed in RSA patients. Therefore, further studies with more patients and controls are required to confirm this result.

The median frequency of Vδ2 cells in peripheral γδ T cells is 74.6% (range 4.5%–90.6%), 84.3% (range 20.0%–96.4%), 82.4% (range 42.9%–90.5%), and 79.7% (range 50.0%–94.0%) in the RSA-P, HC-P, RSA-UP, and HC-UP groups, respectively (**Figure 1B**), which is in accordance with previous publications that Vδ2 cells are the predominant subset of peripheral γδ T cells. We failed to find any difference between the four groups. However, we did find that the differentially expressed markers (i.e., PD1, CD107a, IL-4, and IL-17A) were only observed on Vδ2 cells but not on Vδ1 cells. Previous measurements of these markers were mostly limited to total γδ T cells, and given the high frequency of Vδ2 cells in peripheral γδ T cells, our results are therefore not surprising and are in agreement with previous findings in examining total γδ T cells. Of note, we failed to find any differential expression of aforementioned markers among four groups on Vδ1 cells, the minor population of peripheral γδ T cells. These cells generally exhibited higher expression of PD1, perforin, and IL-17A, as well as lower IFNγ and TNFα expression than Vδ2 cells in our measurement (**Supplementary Figures S6A–E**). Interestingly, the differential capability in producing cytokines (i.e., IL-17A, IFNγ, and TNFα) between Vδ1 cells and Vδ2 cells in RSA-UP, HC-P, and HC-UP groups disappeared in the RSA-P group, indicating that except for Vδ2 cells, the role of Vδ1 cells in pregnancy should be further studied in future investigations.

The presence of γδ T cells at the feto-maternal interface is not new. It was reported that the γδ T cells are present in the endometrium of all mammals throughout pregnancy (14) and in the decidua of human (13), mouse (67), and sheep (73). Decidual γδ T cells were found to be dividing (74) and undergoing TCR recombination (73, 75, 76). Moreover, the number of γδ T cells was growing with an activated phenotype (15, 73, 77, 78) during pregnancy, suggesting a protective role of decidual γδ T cells in pregnancy. However, the abundance of γδ T cells in decidua was not clear, with one study showing that there are numerous γδ T cells making up 60% of all T cells (13), and the other one indicating the rare presence of these cells (79). We used the same method as the latter report (i.e., IHC) and found that there were only a few γδ T cells in both RSA patients and healthy subjects with pregnancy. The discordance could be partially explained by the high sensitivity of the TCR chain to fixation and freezing (74), which also prevented us from detecting Vδ1 cells and Vδ2 cells. Alternatively, RNA sequencing and qRT-PCR were used to detect γδ T cells and TCRδ-defined subsets, and no difference was found between the RSA-P and HC-P groups in terms of the frequencies of TCR-γ, -δ, -δ1, -δ2, and -δ3 chains detected. However, we found that *CCL8*, a gene coding a chemokine with the capacity to recruit an array of inflammatory cells including T cells and macrophages, was upregulated in RSA-P patients compared to HC-P subjects. In addition, CIBERSORT analysis also revealed the increased infiltration of CD8^+^ T cells and M2 macrophages in RSA-P patients. Taken together, these results suggest an inflammatory environment in the feto-maternal interface during early pregnancy in RSA patients. The involvement of γδ T cells in this scenario is currently not clear, although it was reported that γδ T cells could secrete high amount of CCL8 (34, 35) and also can be recruited by CCL8 through expressing corresponding receptors such as CCR1, CCR2, CCR3, and CCR5 (36–40). Therefore, the increased number of CD107a^+^ γδ T cells may also be implicated in CCL8-mediated inflammatory response in RSA either as secreting cells or as responder cells. Further studies utilizing mIHC and/or multicolor flow cytometry and an *in vitro* functional study are required to address this speculation.

In conclusion, through examining peripheral γδ T cells/subsets and their phenotype, we found the increased expression of PD1 and CD107a, as well as higher frequency of IL-17A-secreting cells and lower frequency of IL-4-secreting cells in the compartment of Vδ2 cells in RSA patients during pregnancy, suggesting that the activated phenotype, increased cytotoxicity, and imbalanced cytokine profiles of peripheral Vδ2 cells may lead to the occurrence of RSA. Furthermore, increased CD107a^+^ γδ T cells could be detected in the decidua of RSA-P patients compared to that in the HC-P subjects, accompanied by upregulated *CCL8* expression, as well as increased CD8^+^ T cells and M2 macrophages infiltration, suggestive of the contribution of decidual γδ T cells to the local inflammatory response in RSA. Thus, dysregulated γδ T cells in the periphery and decidua might be a hallmark of RSA; future investigations along this line will provide insight into the role of these cells in the pathogenesis of RSA, and will eventually lead to the successful management of RSA patients by manipulating these cells.

## Data Availability Statement

The datasets presented in this study can be found in online repositories. The names of the repository/repositories and accession number(s) can be found below: NCBI GEO, accession no: GSE178535.

## Ethics Statement

The studies involving human participants were reviewed and approved by the institute ethics committee of Tongji Hospital (Ref. No. TJ-C20180201). The patients/participants provided their written informed consent to participate in this study.

## Author Contributions

YH, WZ, QW, HW, LY, and YaZ designed the study and wrote the manuscript. YaZ and LY contributed equally to this work. In detail, YaZ is responsible for collecting the samples and performing flow cytometry. LY took the responsibilities for analyzing the FACS result and performing the IHC. JX, JL, YiZ, QK, and PZ participated in collecting the samples and clinical information. All authors critically reviewed the manuscript and made key contributions to the analysis and interpretations of the results. All authors contributed to the article and approved the submitted version.

## Funding

This study was financially supported by the Innovative Foundation of Huazhong University of Science and Technology (3004510131) to YH.

## Conflict of Interest

The authors declare that the research was conducted in the absence of any commercial or financial relationships that could be construed as a potential conflict of interest.

## Publisher’s Note

All claims expressed in this article are solely those of the authors and do not necessarily represent those of their affiliated organizations, or those of the publisher, the editors and the reviewers. Any product that may be evaluated in this article, or claim that may be made by its manufacturer, is not guaranteed or endorsed by the publisher.
