# Supplementary material for: Activated γδ T Cells With Higher CD107a Expression and Inflammatory Potential During Early Pregnancy in Patients With Recurrent Spontaneous Abortion
Source: Front Immunol. 2021 Aug 17;12:724662. doi: 10.3389/fimmu.2021.724662 (PMC8416064; doi:10.3389/fimmu.2021.724662)
Supplement: Supplementary file 1 [file DataSheet_1.docx]

Supplementary Material

# Supplementary tables

**Supplementary Table 1** Antibodies, conjugated fluorophores, dilutions, host species, clone, and the providing company used in this study.

| Antibodies | Fluorophore | Dilution | Host Species | Clone | Company |
| --- | --- | --- | --- | --- | --- |
| Anti-human-CD3 | APC/Cy7 | 1:100 | Mouse | SK7 | BD Pharmingen |
| Anti-human-TCRγ/δ | PC5 | 1:100 | Mouse | IMMU510 | Beckman |
| Anti-human-Vδ1 | APC | 1:100 | Mouse | TS8.2 | Invitrogen |
| Anti-human-Vδ1 | Percp/Vio700 | 1:100 | REAfinity | REA173 | Miltenyi |
| Anti-human-Vδ2 | PE/Vio770 | 1:100 | REAfinity | REA771 | Miltenyi |
| Anti-human-PD-1 | BV421 | 1:100 | Mouse | EH12.1 | BD Horizon |
| Anti-human-CD107a | BV421 | 1:100 | Mouse | H4A3 | BD Horizon |
| Anti-human-perforin | AF647 | 1:100 | Mouse | δG9 | BD Pharmingen |
| Anti-human-Granzyme B | PE | 1:100 | Mouse | GB11 | BD Pharmingen |
| Anti-human-IFNγ | PE | 1:20 | Mouse | B27 | BD Pharmingen |
| Anti-human-TNFα | PE | 1:20 | Mouse | MAb11 | BD Pharmingen |
| Anti-human-IL-4 | APC | 1:50 | Rat | MP4-25D2 | BD Pharmingen |
| Anti-human-IL-17A | AF647 | 1:50 | Mouse | SCPL1362 | BD Pharmingen |
| Anti-human-TCRγ/δ | NA | 1:150 | Mouse | H-41 | Santa Cruz Biotech |
| Anti-human-CD107a | NA | 1:100 | Mouse | H4A3 | BD Pharmingen |
| Anti-mouse -IgG | HRP | 1:200 | Goat | NA | Akoya Biosciences |
| Opal 520 Reagent | NA | 1:100 | NA | NA | Akoya Biosciences |
| Opal 620 Reagent | NA | 1:100 | NA | NA | Akoya Biosciences |

**Supplementary Table 2** Primers used for qRT-PCR in this study

| **primers** | **source** | **Sequence (3’-5’)** |
| --- | --- | --- |
| h*CCL8*-F | Tsingke Biotechnology | TGGAGAGCTACACAAGAATCACC |
| h*CCL8*-R | Tsingke Biotechnology | TGGTCCAGATGCTTCATGGAA |
| h*TRDV1*-F | Tsingke Biotechnology | TCCATGCCAGTGAGGAAAGC |
| h*TRDV1*-R | Tsingke Biotechnology | GTTAAGGCGACGGATTTCGC |
| h*TRDV2*-F | Tsingke Biotechnology | CCAAGCAAGACTCAGCAGCA |
| h*TRDV2*-R | Tsingke Biotechnology | ACCCCTATTGACACAGGCAC |
| h*TRDV3*-F | Tsingke Biotechnology | GCTGTACTCACTGTCTGGGG |
| h*TRDV3*-R | Tsingke Biotechnology | AAGTGCAGAGCAGTACCACC |
| h*GAPGH*-F | Tsingke Biotechnology | CCATCAATGACCCCTTCATTGACC |
| h*GAPGH*-R | Tsingke Biotechnology | GAAGGCCATGCCAGTGAGCTTCC |

# Supplementary Figures legends

**Figure S1. Related to Figure 1. The frequencies of T cell subsets and their PD1 expression in the peripheral blood of RSA patients and healthy controls with or without pregnancy**. Peripheral blood from four groups were stained for T cell subset and PD1 expression. **(A)** Gating strategy used for analysis of T cell subsets and PD1 expression. (**B**) The frequencies of Vδ1^+^ cells, Vδ2^+^ cells and Vδ1^-^Vδ2^-^ cells in γδ T cells. Left, representative flow cytometry plots; Right, statistical data show means ± s.e.m. (**C**) The ratios of different γδ T cell subsets in all T cells. (**D**) The ratios of different γδ T cell subsets in γδ T cells. (**E**) PD1 expression on Vδ1^+^ cells, Vδ2^+^ cells and Vδ1^-^Vδ2^-^ cells gated from γδ T cells. Statistical analyses using Mann–Whitney U-test for the same cells among different groups. RSA-P, RSA pregnant, n=10; HC-P, healthy pregnant, n=11; RSA-UP, RSA unpregnant, n=11; HC-UP, healthy unpregnant, n=10.

**Figure S2. Related to Figure 2. The frequencies of perforin^+^GZMB^+^ cells in peripheral γδ T cells in RSA patients and healthy controls with or without pregnancy.** PBMCs were obtained from peripheral blood of each subject and directly stained for membrane CD107a as well as intracellular perforin and GZMB. **(A)** Gating strategy used for analysis of perforin, GZMB, and CD107a expression on γδ T cell subsets. (**B**) The frequencies of perforin^+^GZMB^+^ cells in Vδ1^+^ cells and Vδ2^+^ cells from four groups. Statistical data show means ± s.e.m. Statistical analyses using Mann–Whitney U-test among different groups. Difference are indicated: * *p* < 0.05. RSA-P, RSA pregnant, n=10; HC-P, healthy pregnant, n=11; RSA-UP, RSA unpregnant, n=11; HC-UP, healthy unpregnant, n=10.

**Figure S3. Related to Figure 3. The cytokine profiles of Vδ1^+^ and Vδ2^+^ γδ T cells in the peripheral blood of RSA patients and healthy controls with or without pregnancy**. PBMCs were obtained from peripheral blood of each subject and stimulated with PMA/Ionomycin/ Brefeldin A for 5 hours before being stained for intracellular cytokines. **(A)** Gating strategy used for analysis of TNFα, IFNγ, IL-4 and IL-17A expression on γδ T cell subsets. (**B**) The frequencies of IFNγ-secreting cells in Vδ1^+^ cells and Vδ2^+^ cells. (**C**) The frequencies of TNFα-secreting cells in Vδ1^+^ cells and Vδ2^+^ cells. Left, representative flow cytometry plots; Right, statistical data show means ± s.e.m. Statistical analyses using Mann–Whitney U-test among different groups. RSA-P, RSA pregnant, n=10; HC-P, healthy pregnant, n=11; RSA-UP, RSA unpregnant, n=11; HC-UP, healthy unpregnant, n=10.

**Figure S4. Related to Figure 4. Transcriptomic analysis of decidual samples from RSA-P patients and HC-P subjects.** Bulk RNA-seq was performed with decidual samples from RSA-P patients (n=3) and HC-P subjects (n=3). **(A)** PCA plot for transcriptomic data. (**B**) KEGG analysis for DEGs. (**C**) CIBERSORT analysis for the immune cells, in which CD8^+^ T and M2 cells were obviously increased in RSA group (right). Statistical data show means ± s.e.m. Statistical analyses using Mann–Whitney U-test among different groups. Difference are indicated: * *p* < 0.05.

**Figure S5. Related to Figure 5. TCR γδ expression level and the presence of CD107a^+^γδ T cells in the decidua of pregnant RSA patients. (A)** The numbers of BCR and TCR chains recovered from transcriptomic data. (**B**) qRT-PCR analysis for *TRDV1*, *TRDV2*, and *TRDV3* expression in RSA-P and HC-P decidua with 14 samples from each group. (C) The frequencies of CD107a^+^ cells in decidual γδ T cell with 4 samples from each group determined by mIHC. Statistical data show means ± s.e.m. Statistical analyses using Mann–Whitney U-test among different groups. Difference are indicated: * *p* < 0.05.

**Figure S6. Referred in Discussion section. The frequencies of PD1^+^, perforin^+^, IL-17A^+^, IFNγ^+^ and TNFα^+^ cells in Vδ1^+^ cells and Vδ2^+^ cells in RSA patients and healthy controls with or without pregnancy.** Vδ1^+^ cells and Vδ2^+^ cells were re-compared for the expression of PD1, perforin, IL-17A, IFNγ and TNFα. **(A)** The frequencies of PD1^+^ cells in Vδ1^+^ cells and Vδ2^+^ cells from four groups. (**B**) The frequencies of perforin^+^ cells in Vδ1^+^ cells and Vδ2^+^ cells. **(C)** The frequencies of IL-17A^+^ cells in Vδ1^+^ cells and Vδ2^+^ cells. (**D**) The frequencies of IFNγ^+^ cells in Vδ1^+^ cells and Vδ2^+^ cells. **(E)** The frequencies of TNFα^+^ cells in Vδ1^+^ cells and Vδ2^+^ cells. Statistical data show means ± s.e.m. Statistical analyses using Wilcoxon matched-pairs signed rank test among different groups. Difference are indicated: *** *p* < 0.001, ** *p* < 0.01, and * *p* < 0.05. RSA-P, RSA pregnant, n=10; HC-P, healthy pregnant, n=11; RSA-UP, RSA unpregnant, n=11; HC-UP, healthy unpregnant, n=10.
