# Supplementary figures and images for: Activated γδ T Cells With Higher CD107a Expression and Inflammatory Potential During Early Pregnancy in Patients With Recurrent Spontaneous Abortion
Source: Front Immunol. 2021 Aug 17;12:724662. doi: 10.3389/fimmu.2021.724662 (PMC8416064; doi:10.3389/fimmu.2021.724662)

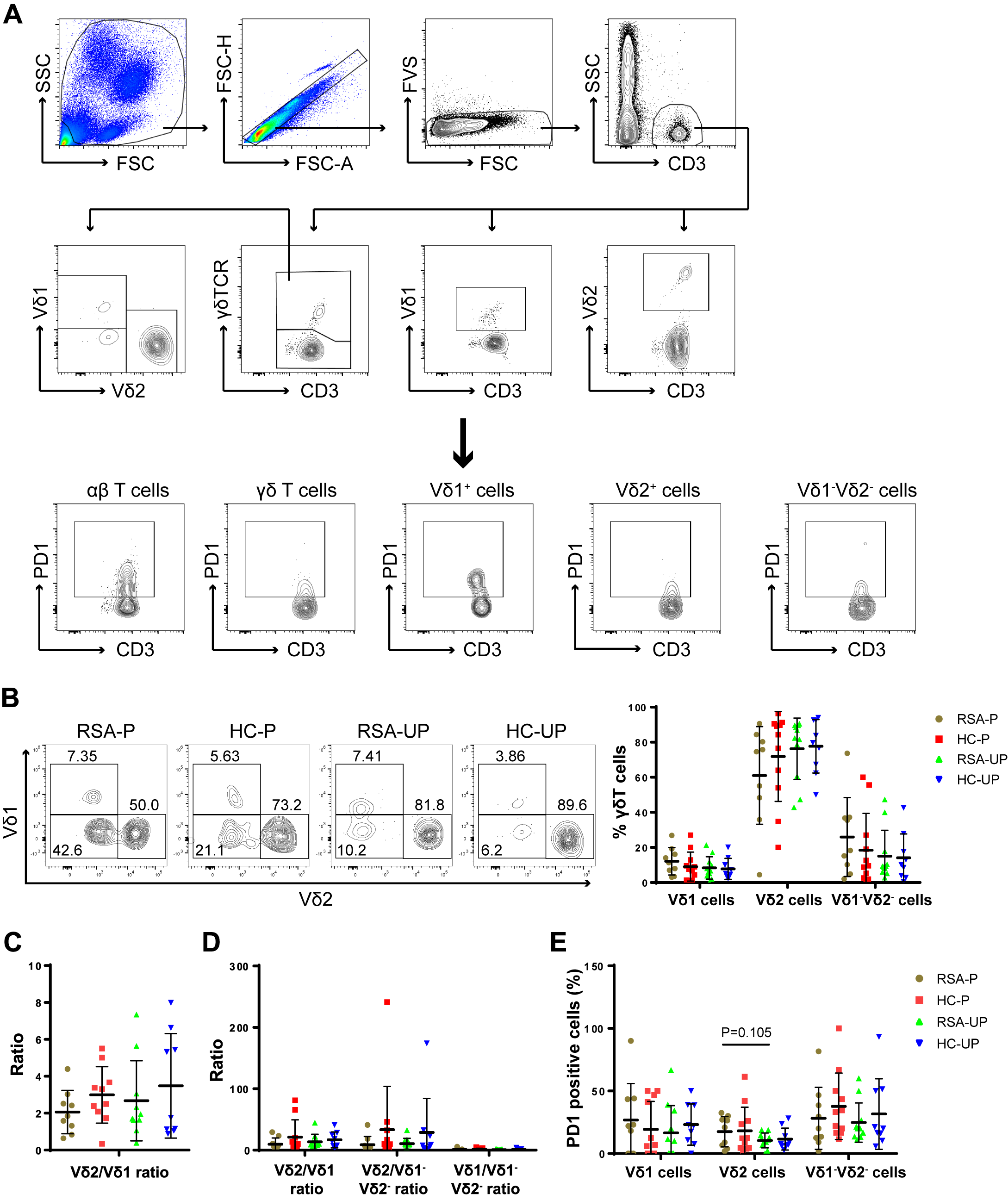

Supplement: Supplementary file 2 [file Image_1.tif]

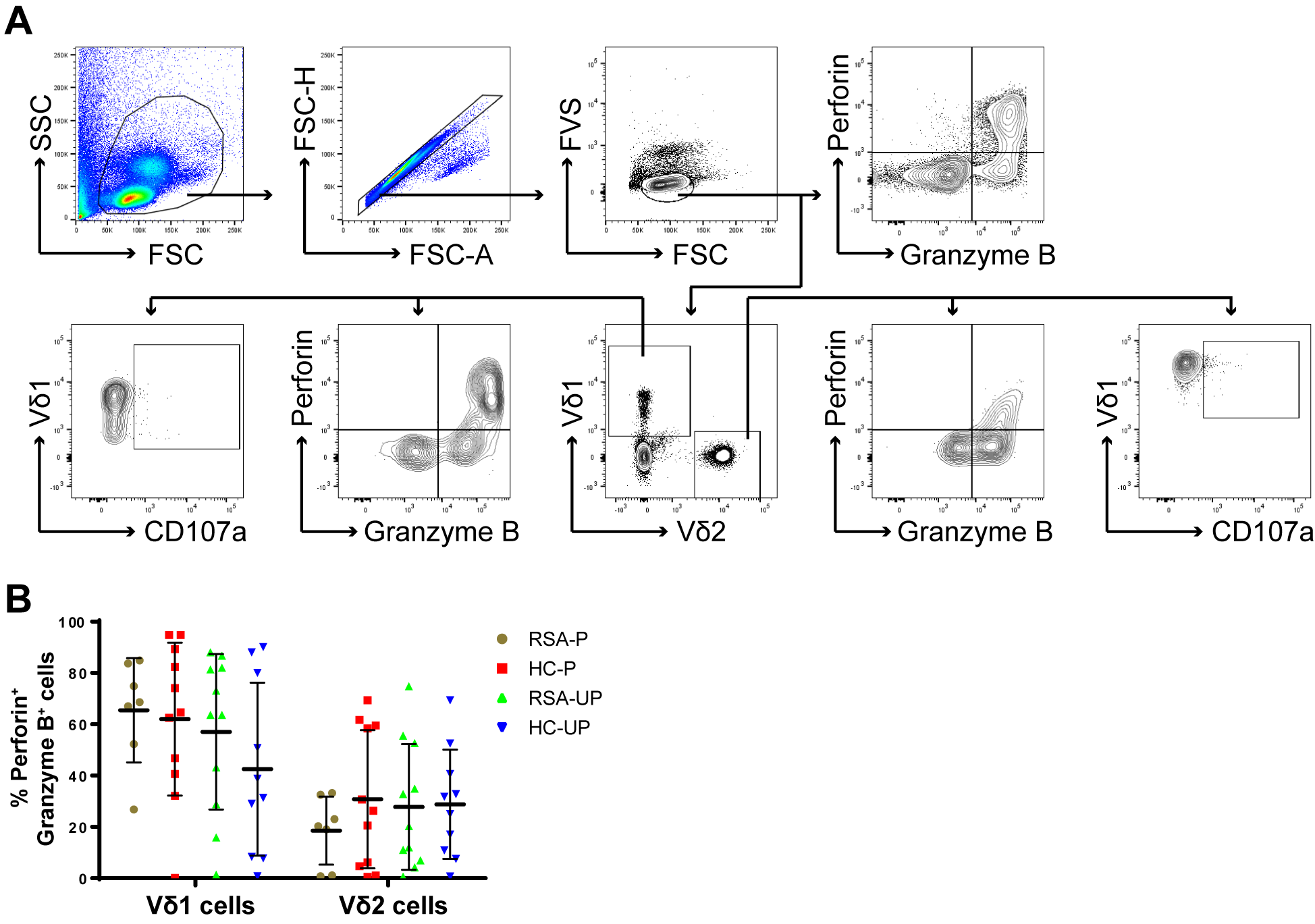

Supplement: Supplementary file 3 [file Image_2.tif]

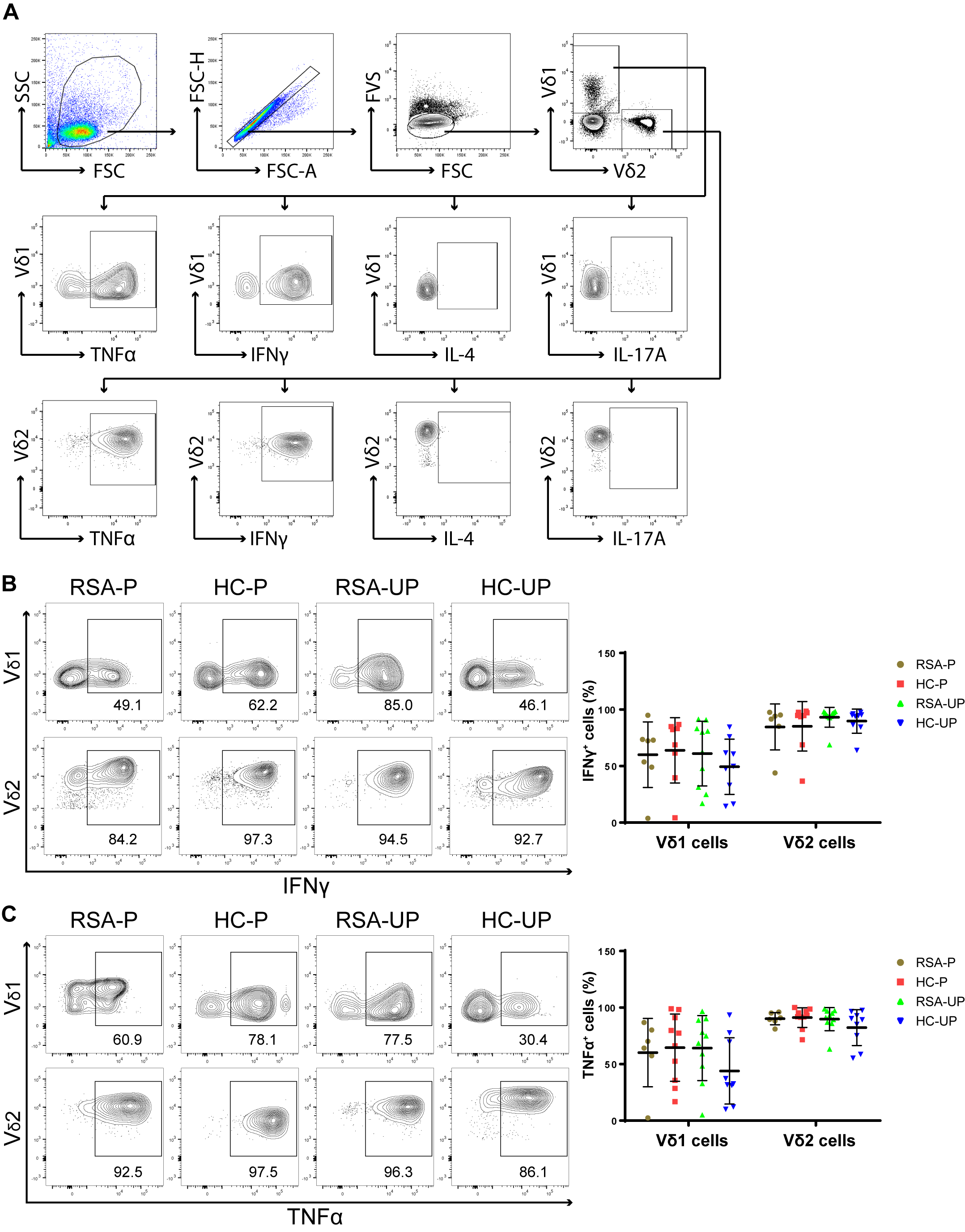

Supplement: Supplementary file 4 [file Image_3.tif]

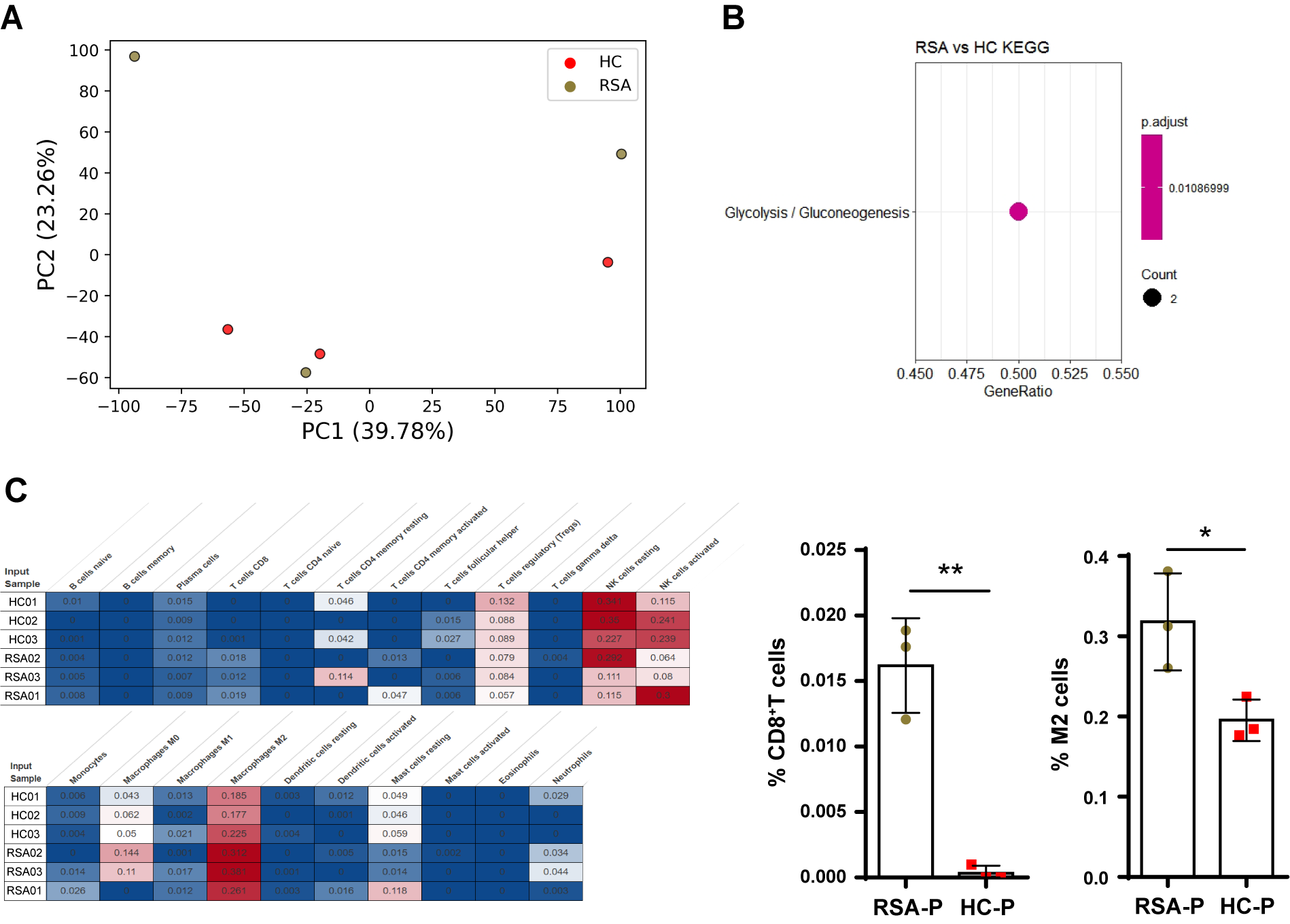

Supplement: Supplementary file 5 [file Image_4.tif]

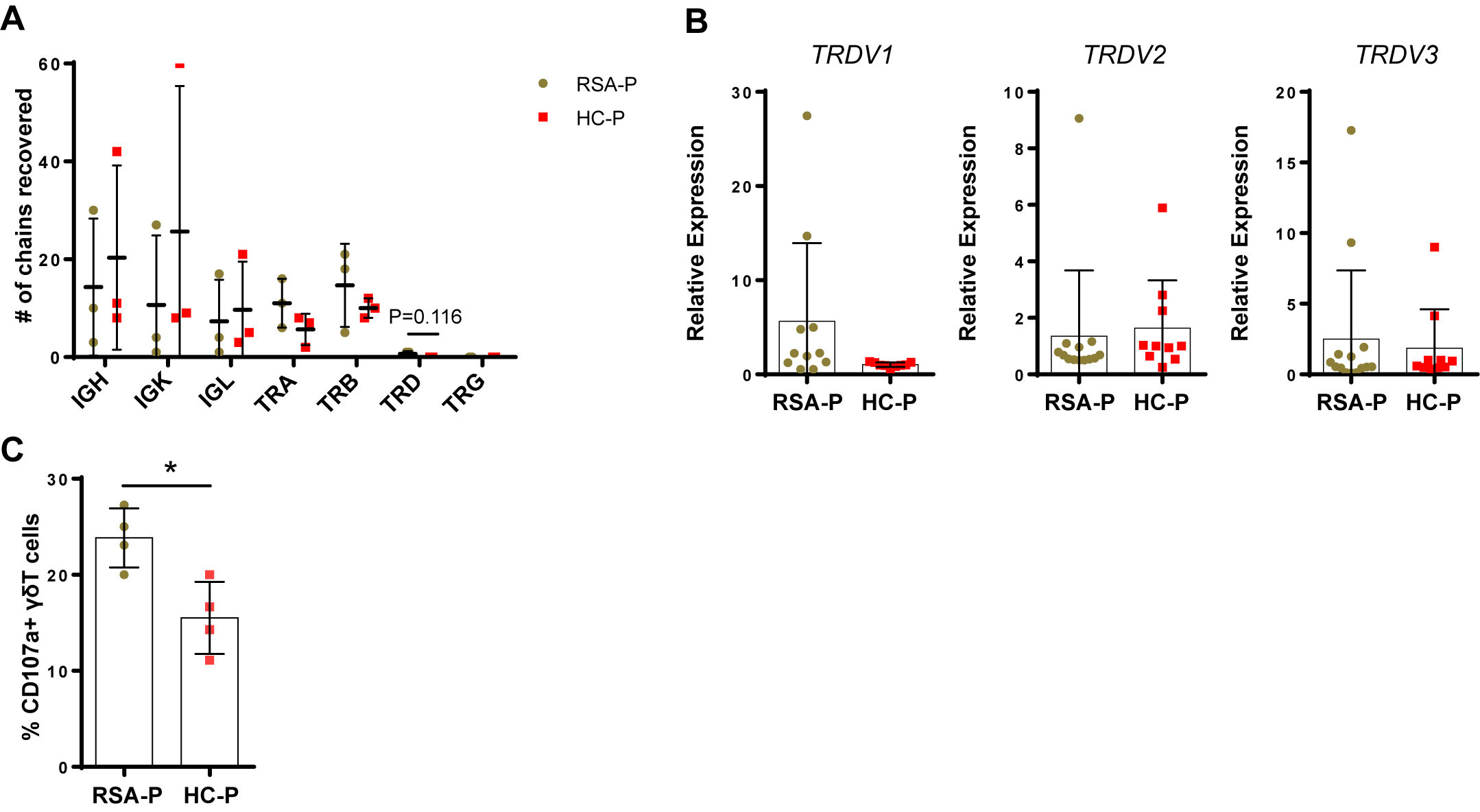

Supplement: Supplementary file 6 [file Image_5.tif]

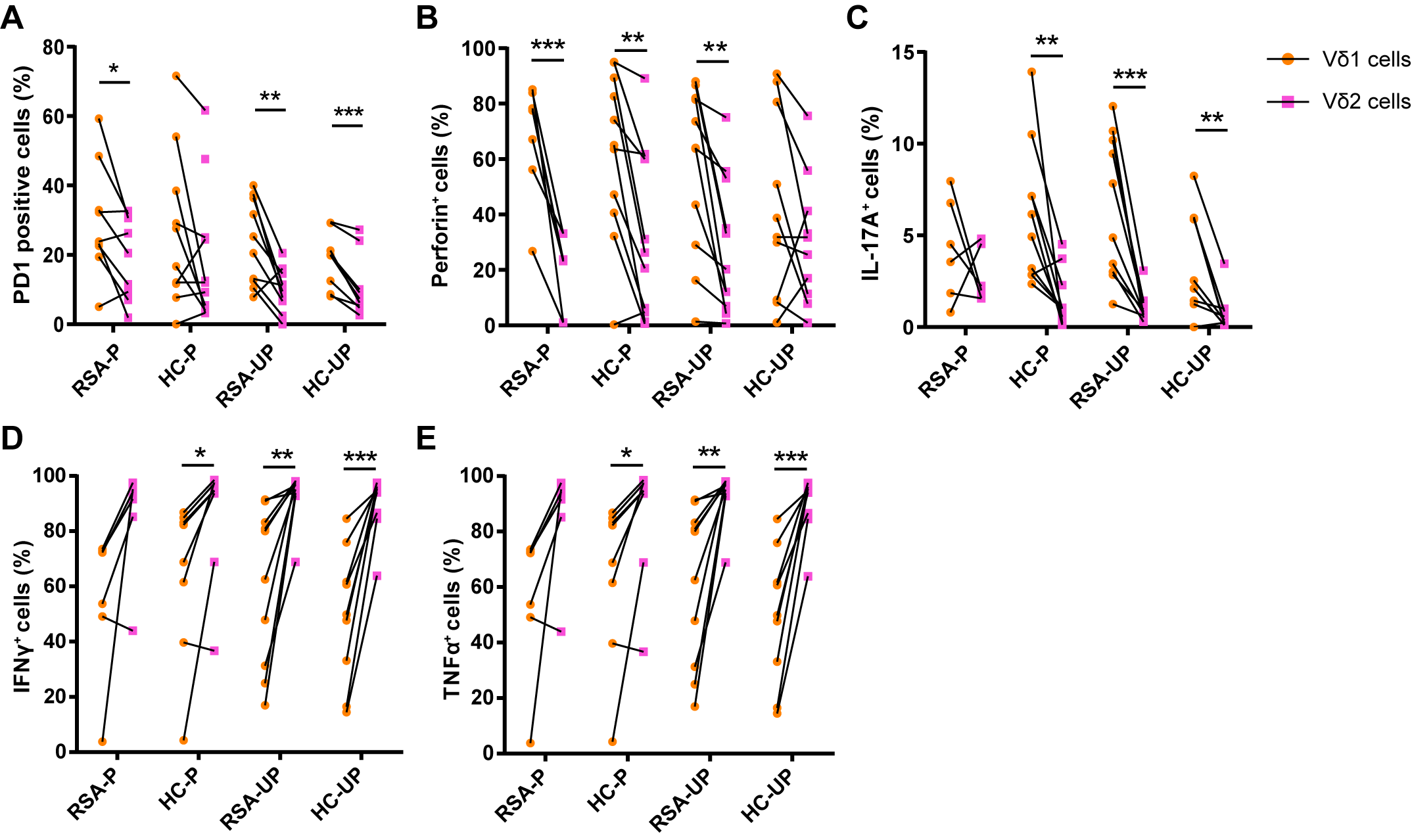

Supplement: Supplementary file 7 [file Image_6.tif]
